# Supplementary material for: Complex transcriptional regulations of a hyperparasitic quadripartite system in giant viruses infecting protists
Source: Nat Commun. 2024 Oct 9;15:8608. doi: 10.1038/s41467-024-52906-1 (PMC11464507; doi:10.1038/s41467-024-52906-1)
Supplement: Supplementary file 1 — Supplementary Information [file 41467_2024_52906_MOESM1_ESM.pdf]

Complex transcriptional regulations of a hyperparasitic quadripartite system in giant viruses infecting protists

Alexandra Bessenay [1], Hugo Bisio [1], Lucid Belmudes [2], Yohann Couté [2], Lionel Bertaux [1,3], Jean-Michel Claverie [1], Chantal Abergel [1], Sandra Jeudy [1,*] and Matthieu Legendre [1,*]

1. Aix-Marseille University, Centre National de la Recherche Scientifique, Information Génomique & Structurale (IGS), Unité Mixte de Recherche 7256 (Institut de Microbiologie de la Méditerranée, FR3479), IM2B, IOM, 13288 Marseille Cedex 9, France.

2. Univ. Grenoble Alpes, INSERM, CEA, UA13 BGE, CNRS, CEA, FR2048, 38000 Grenoble, France.

3. Present address: Aix-Marseille University, Centre National de la Recherche Scientifique, Laboratoire de Chimie Bactérienne (LCB), Unité Mixte de Recherche 7283 (Institut de Microbiologie de la Méditerranée, FR3479), IM2B, 13009 Marseille, France.

* Correspondence: Sandra.Jeudy@igs.cnrs-mrs.fr and legendre@igs.cnrs-mrs.fr

Supplementary Information

This file includes:

Supplementary Figures S1 to S10

Supplementary References

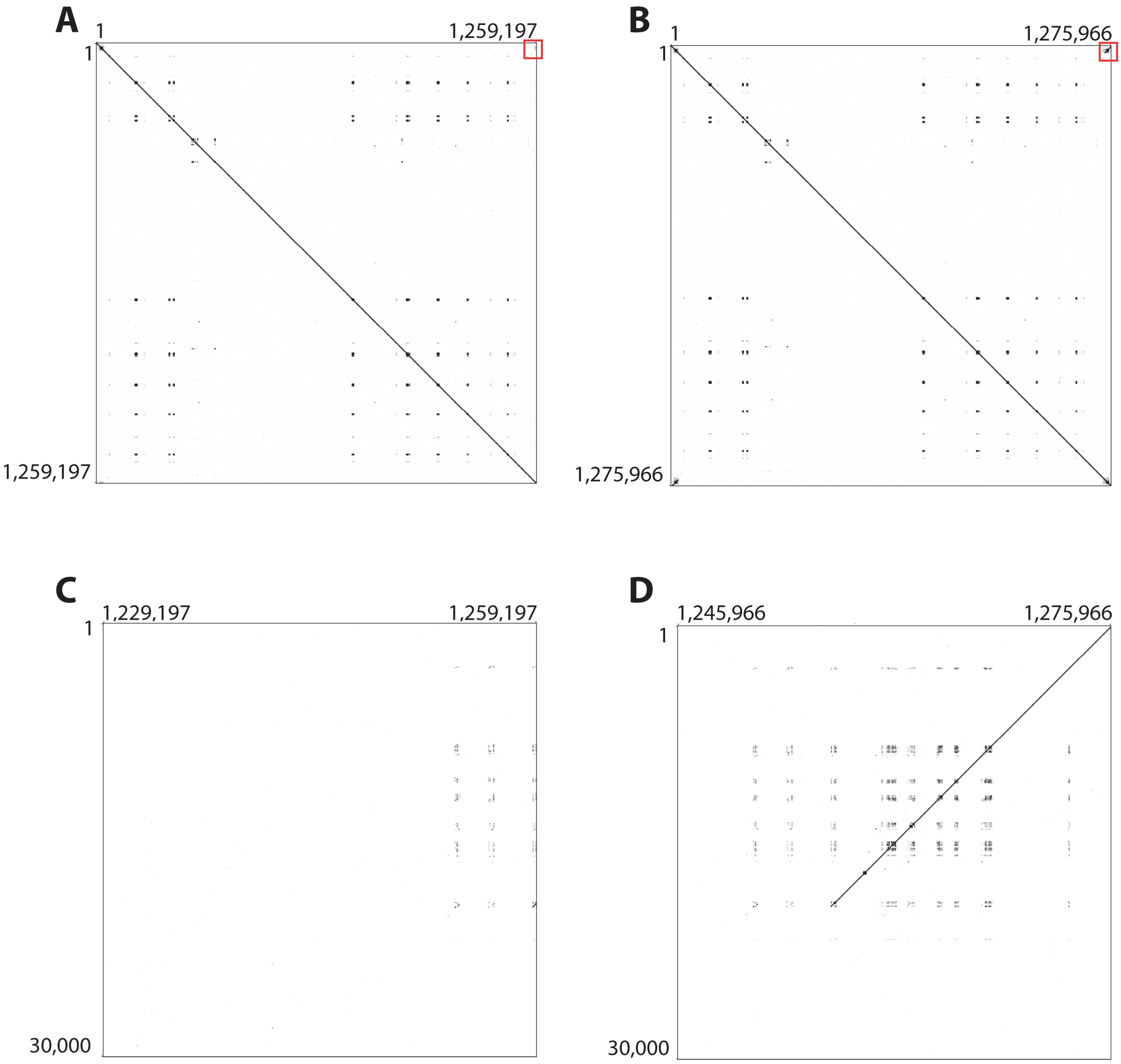

Figure S1. Identification of TIRs in the megavirus chilensis genome

Dot plots of the (A) published [1] and (B) reassembled megavirus chilensis genomes. Dotplots were computed using Gepard [2] with a word length=15. The red boxes on the top right corner of each dotplot indicate the presence or absence of a TIR. Zoomed dotplots of the corresponding regions are shown for the published (C) and reassembled (D) genomes.

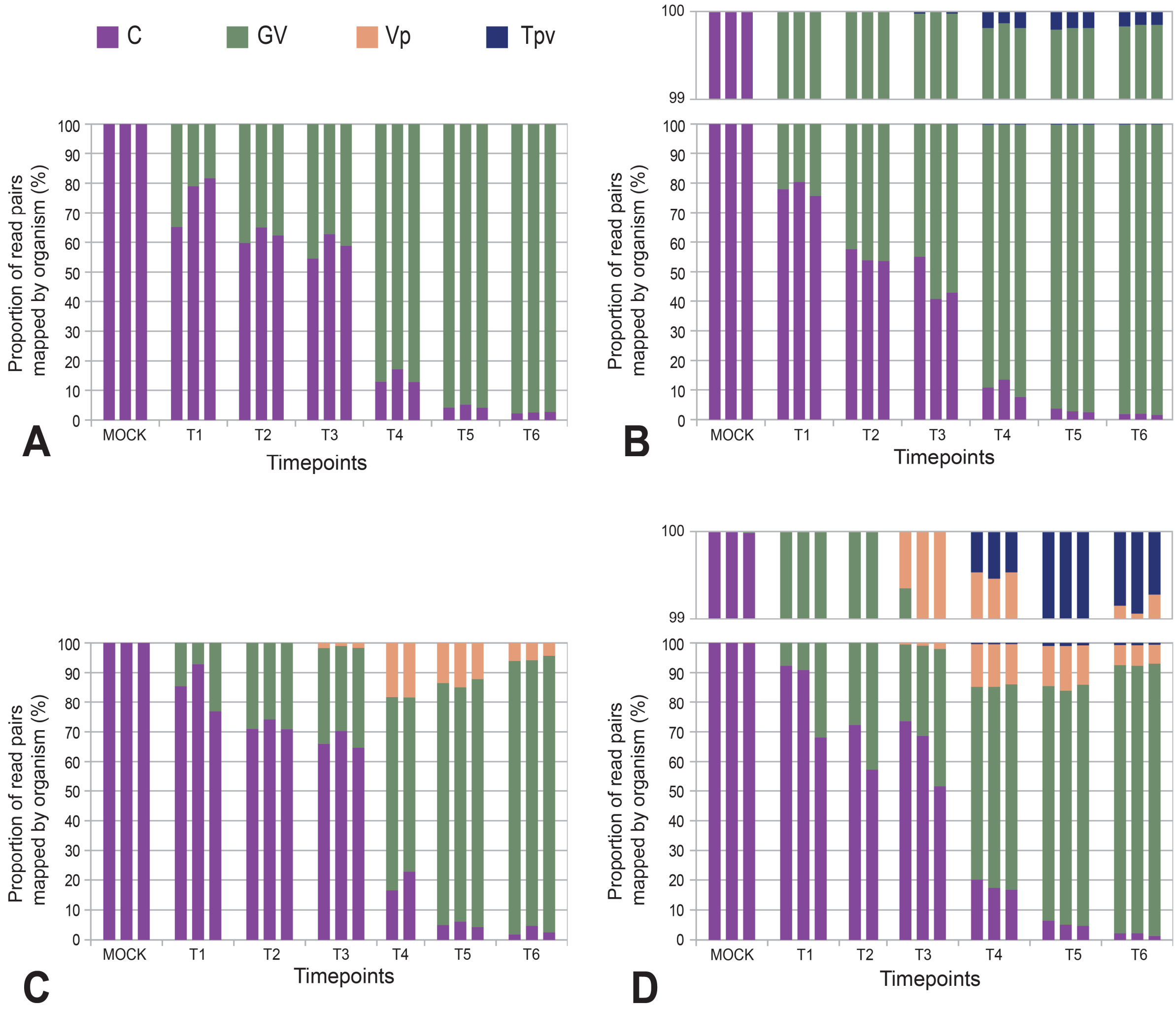

Figure S2. Proportion of read pairs mapped to the reference genomes

Barplots of the proportion of read pairs mapped to each partners' genome (including unique and multi mapped reads). Colors correspond to the different partners (C, GV, Tpv and Vp, Fig. 1A) and labels A-D to the 4 conditions (Fig. 1A): (A) C+GV, (B) C+GV+Tpv, (C) C+GV+Vp and (D) C+GV+Vp+Tpv. Source data are provided as a Source Data file.

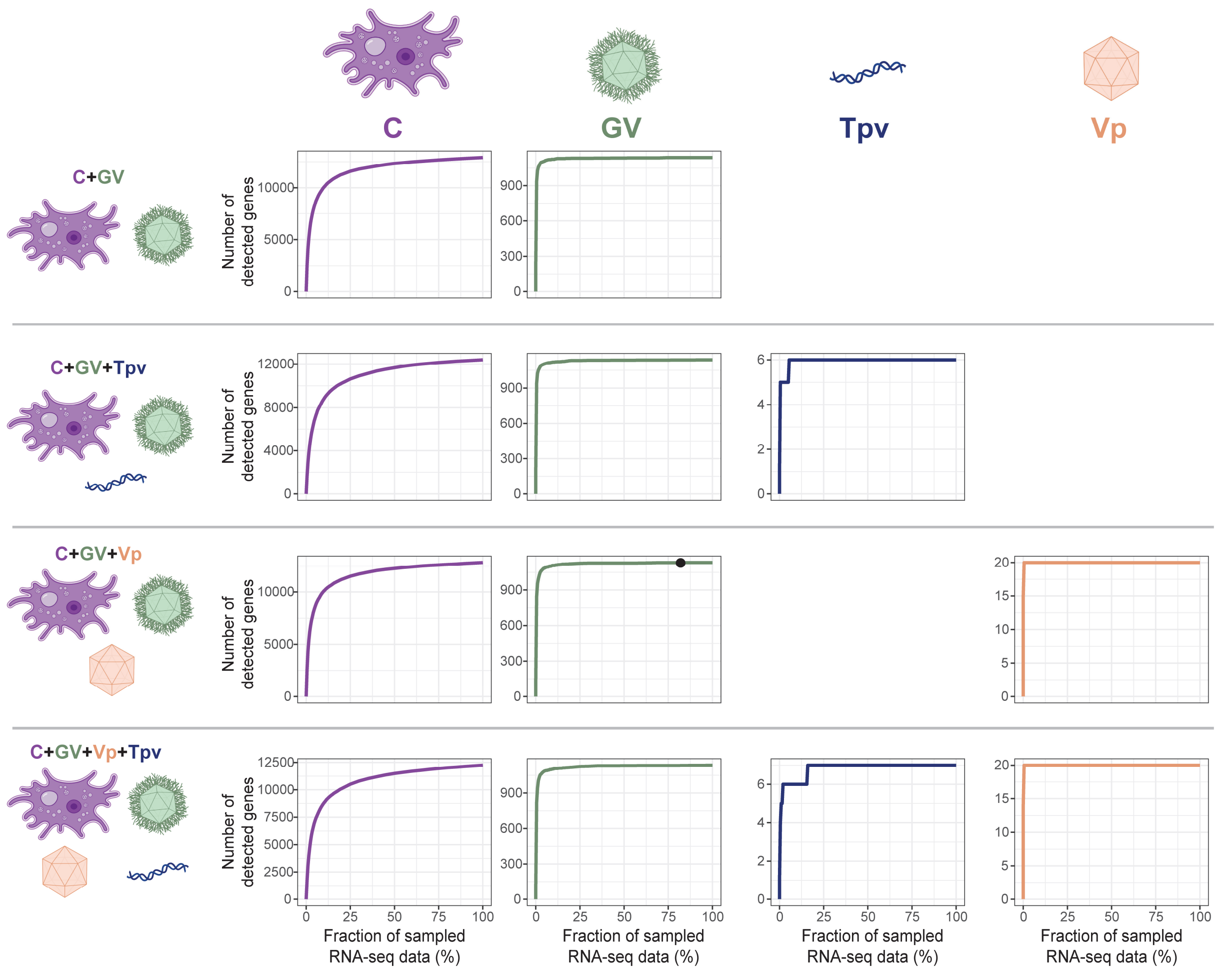

Figure S3. Saturation curves of the partners in all conditions at T4

Saturation curves of the number of genes detected (>5 read pairs per gene) as a function of the percentage of data (from pooled replicates) used for all partners (columns) in all conditions (rows) at T4. RNA-seq data where randomly sampled at 0.01%, 0.5% to 20% (with 0.5% steps) and 25% to 95% (with 5% steps) of the datasets. Black dot on the GV curve in the C+GV+Vp condition shows the full coverage when the Vp genome sequence is excluded from the mapping. Source data are provided as a Source Data file. Icons representing partners were created with BioRender.com.

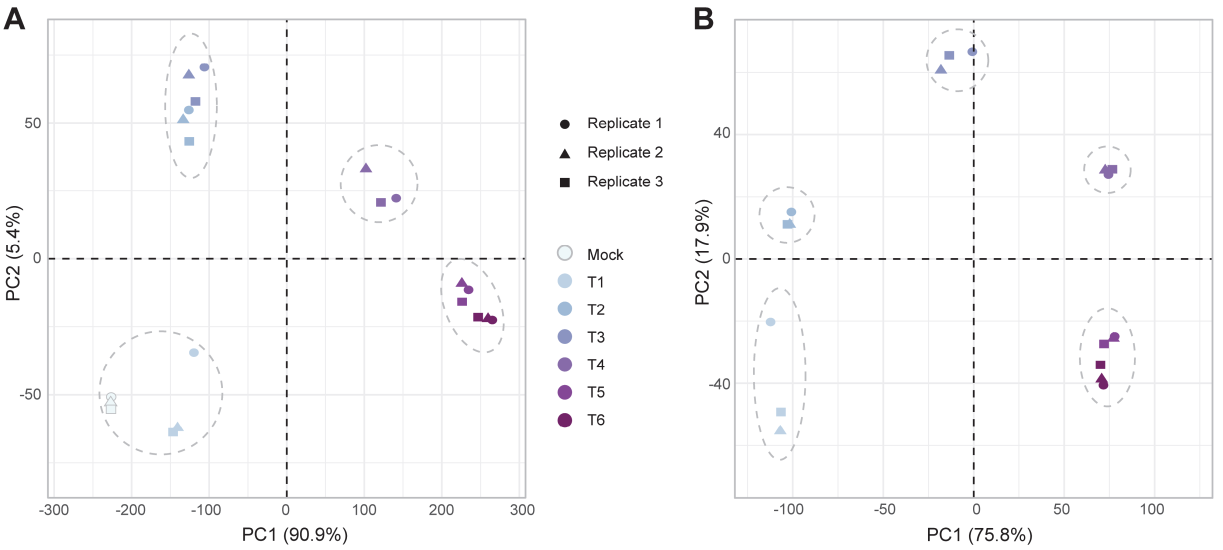

Figure S4. PCA of *A. castellanii*-megavirus chilensis single infection

PCA of host cell (A) and GV (B) TPM gene expression values. The three replicates are depicted with different symbols, while the mock and timepoints are color-coded. Dashed ellipses delimit coarse manual grouping of samples.

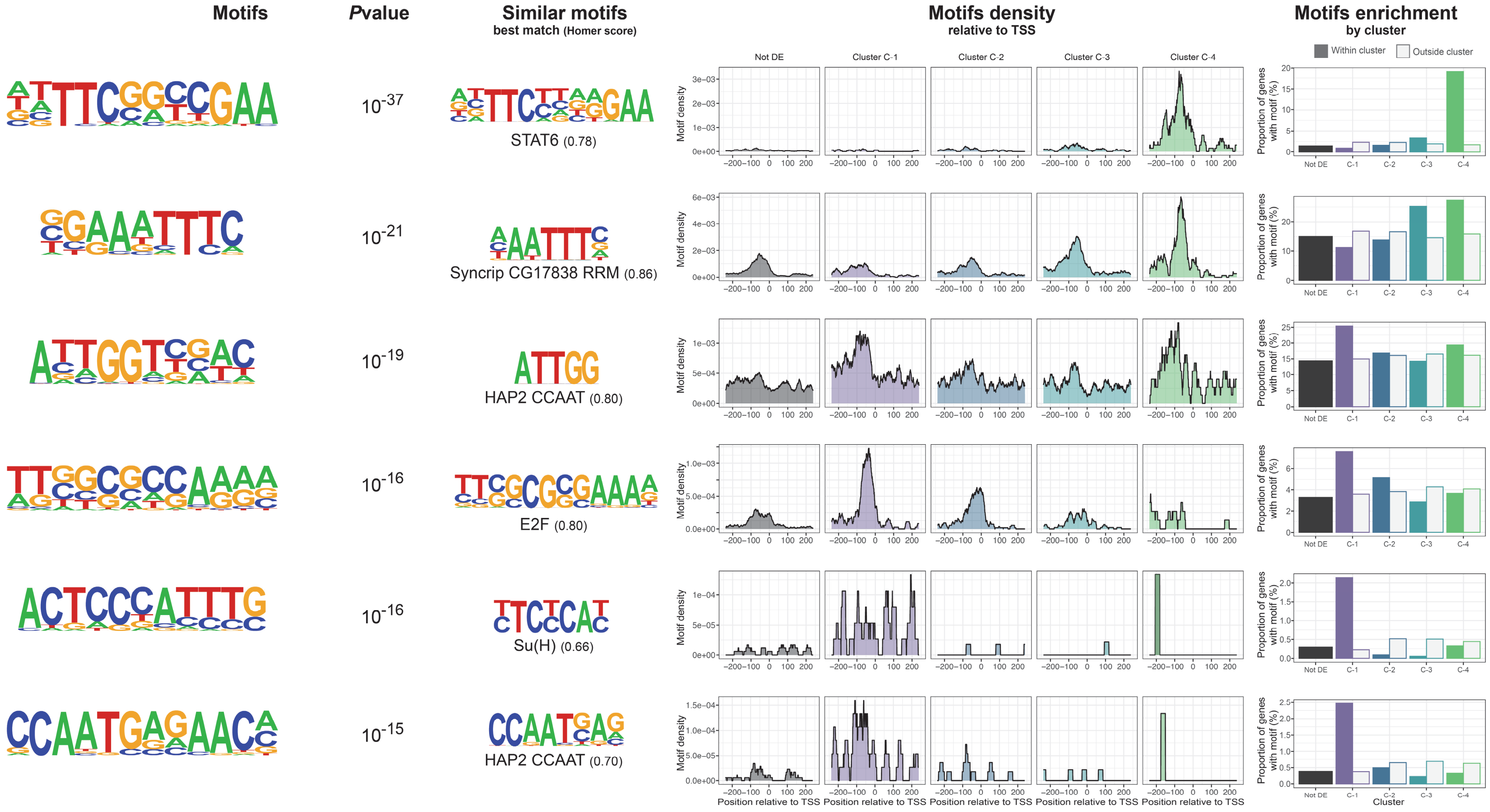

Figure S5. Enriched motifs in *A. castellanii* promoters

Motifs enriched in the vicinity of *A. castellanii* TSS (-250/+250 nt) found using Homer [3]. Motifs' logos are shown on the left with associated enrichment *P*values. Most similar known motifs according to Homer are shown as well. Graphs show the density of each motif in the 4 cellular genes clusters (see Fig. 2) from the C+GV condition, as well as in the group of genes not differentially expressed (Not DE). Graphs on the right show the proportion of promoter sequences that match each motif within (or outside) each cluster.

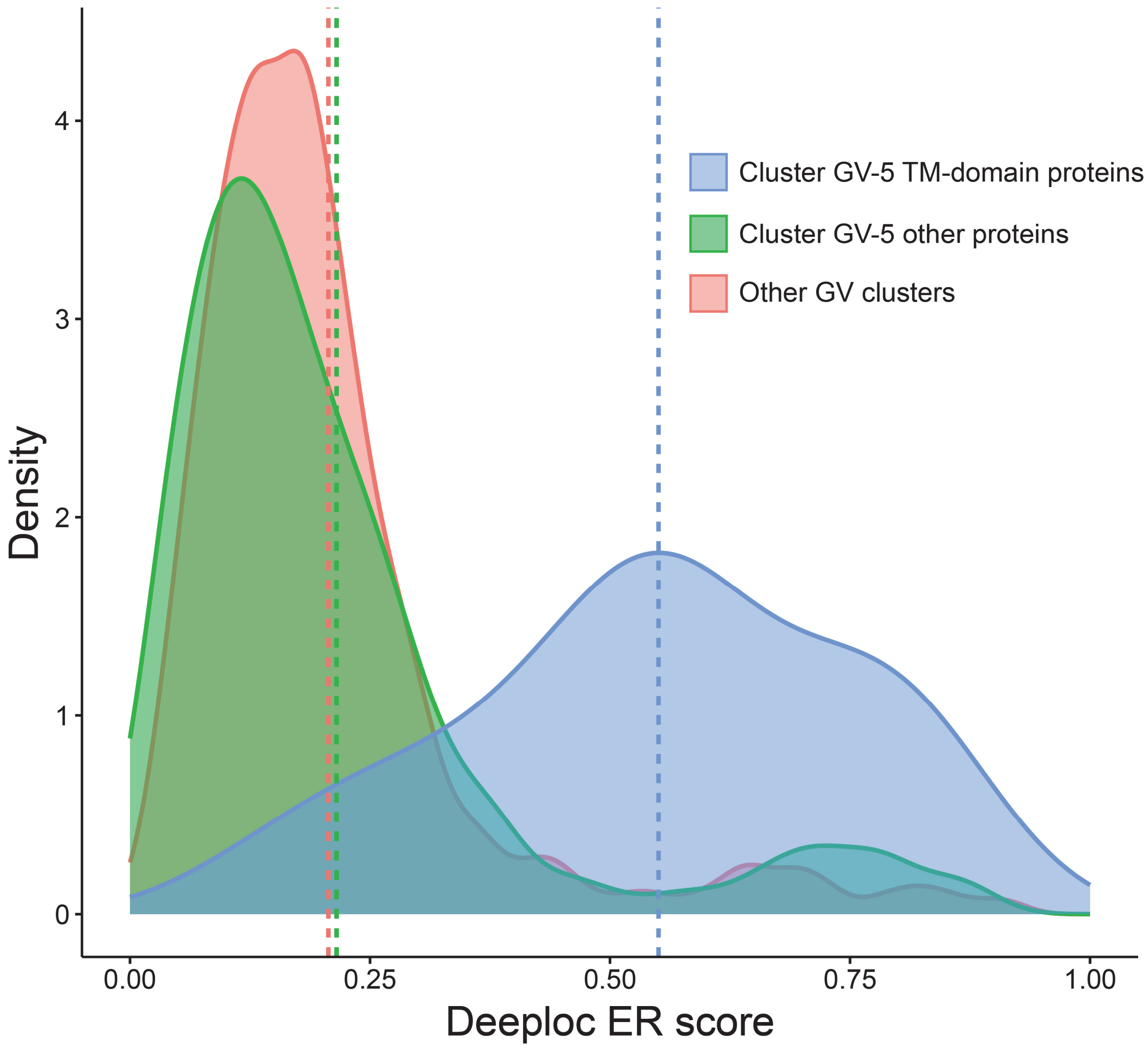

Figure S6. Distribution of DeepLoc ER score of TM-domain proteins compared to other proteins
Shown are the density distributions of the DeepLoc score for predicted localization at endoplasmic reticulum (ER). Genes from cluster GV-5 (Fig. 3A) with a predicted transmembrane (TM)-domain are compared to other proteins. Dashed lines correspond to the mean. Distributions are significantly different (Kruskal-Wallis test *P*value = 8.4×10^{-13}). Source data are provided as a Source Data file.

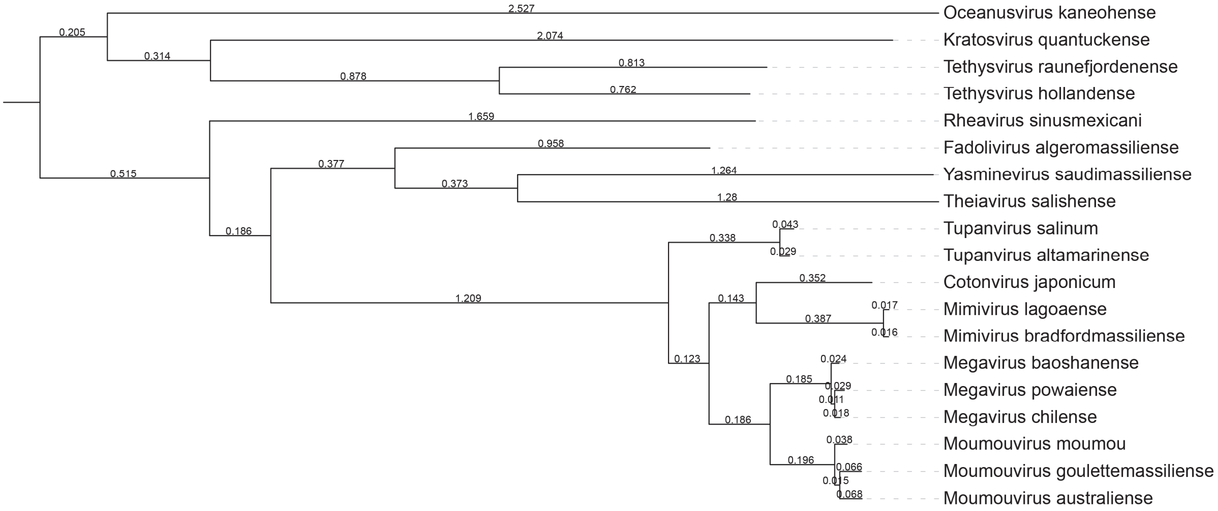

Figure S7. Phylogenetic tree of *Imitervirales*

Phylogenetic tree of *Imitervirales* computed using OrthoFinder [4] from clusters of orthologous genes (see Methods). Branch length is noted above each branch.

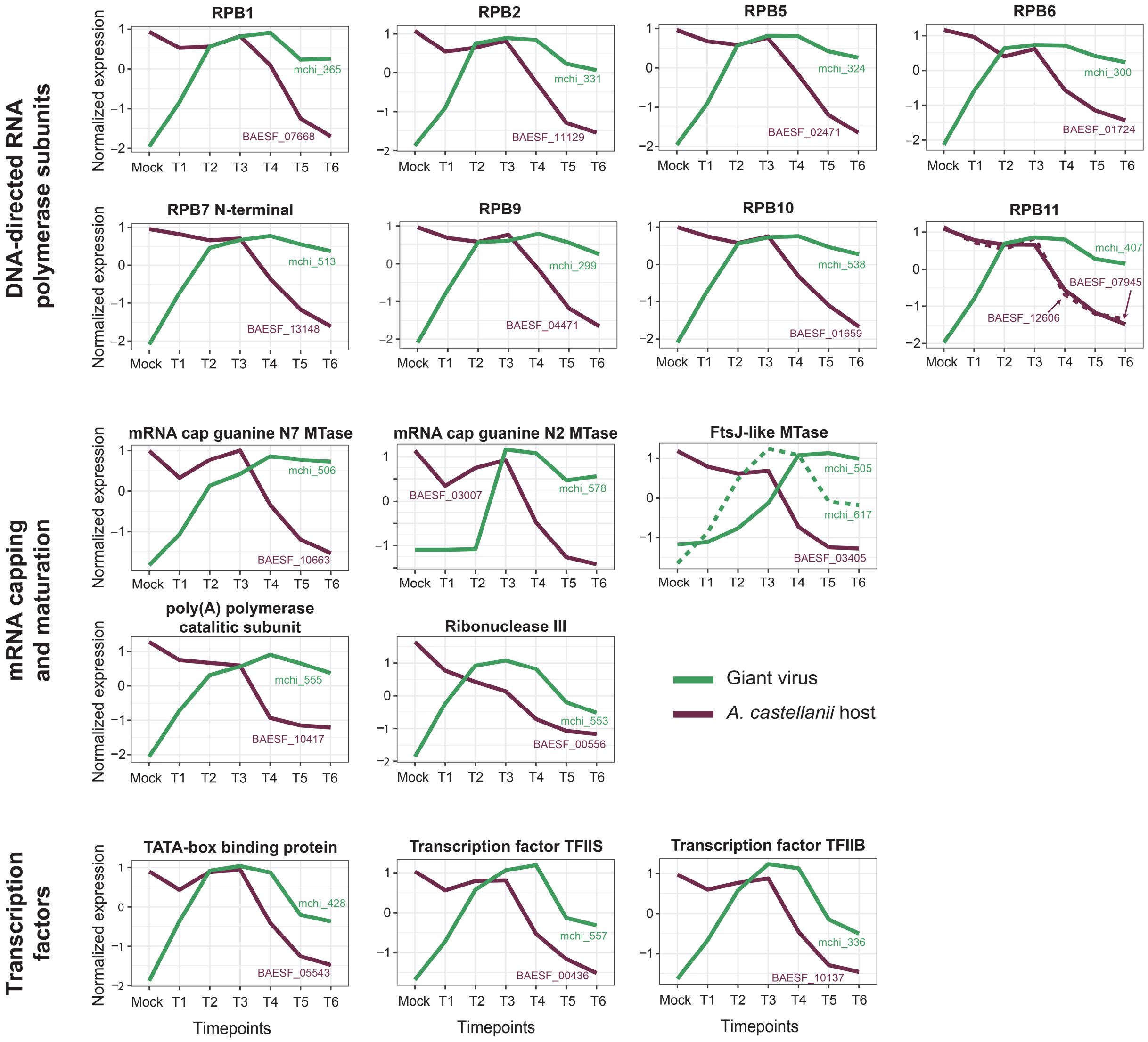

Figure S8. Expression of megavirus chilensis virally-encoded and *A. castellanii* host homologous genes involved in transcription

Gene expression profiles of cellular (purple) and viral (green) homologs involved in identical predicted functions related to transcription are shown. When paralogs are present, the second copy is indicated with a dotted line. Normalized expression corresponds to Z-score of log_2-transformed TPM values. Genes are classified in broad categories (DNA directed RNA polymerase subunits, mRNA capping and maturation, and transcription factors). Source data are provided as a Source Data file.

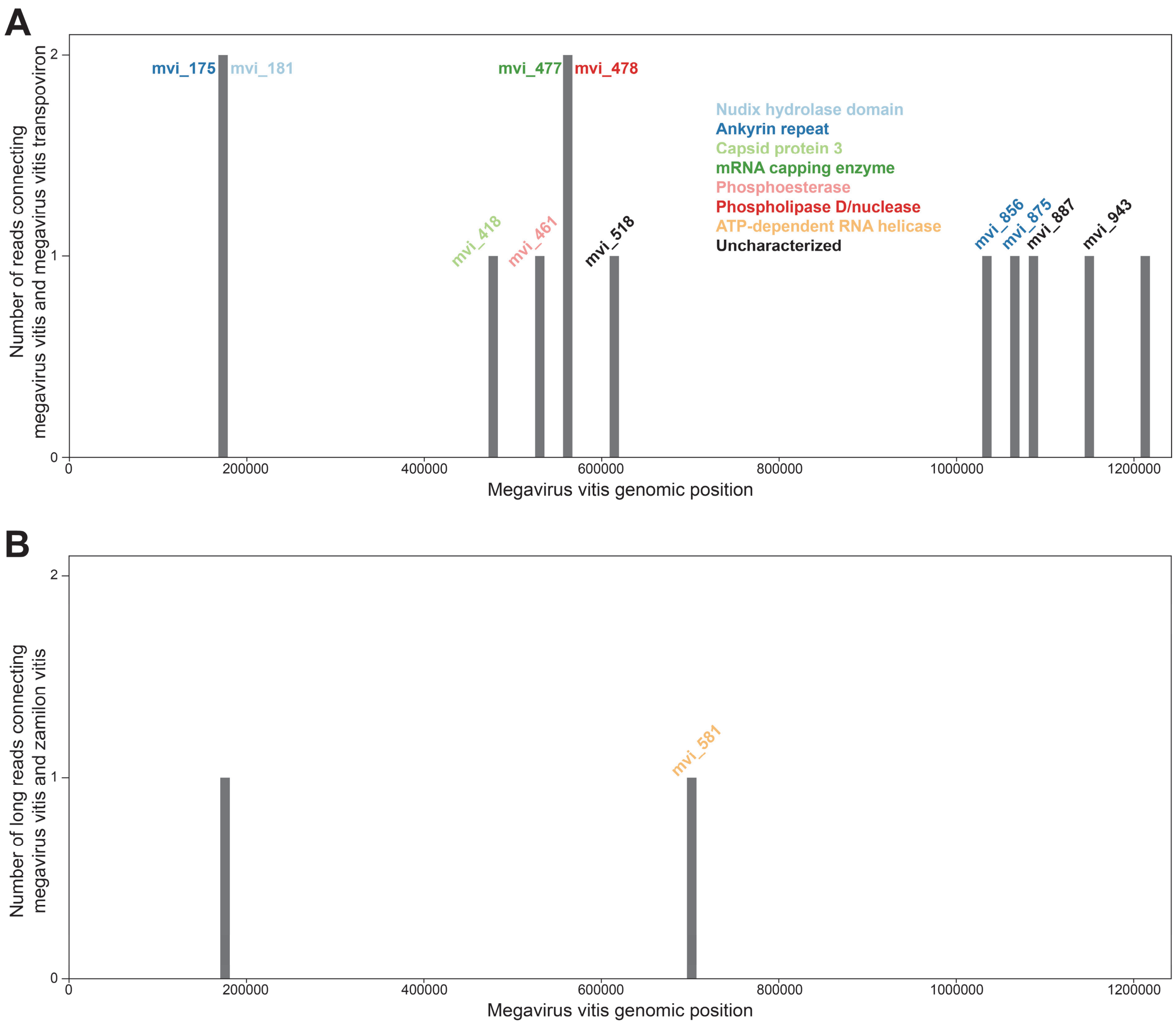

Figure S9. Genomic positions of potential integration of megavirus vitis transpoviron and zamilon vitis in the megavirus vitis genome

Bars correspond to the number of nanopore long reads (y-axis) connecting either megavirus vitis transpoviron (A) or zamilon vitis (B) to megavirus vitis genomic sequence. The genomic locations in the megavirus vitis genome of these potential integration positions are shown on the x-axis. Megavirus vitis genes overlapping these genomic locations and their corresponding annotations (color-coded) are also shown.

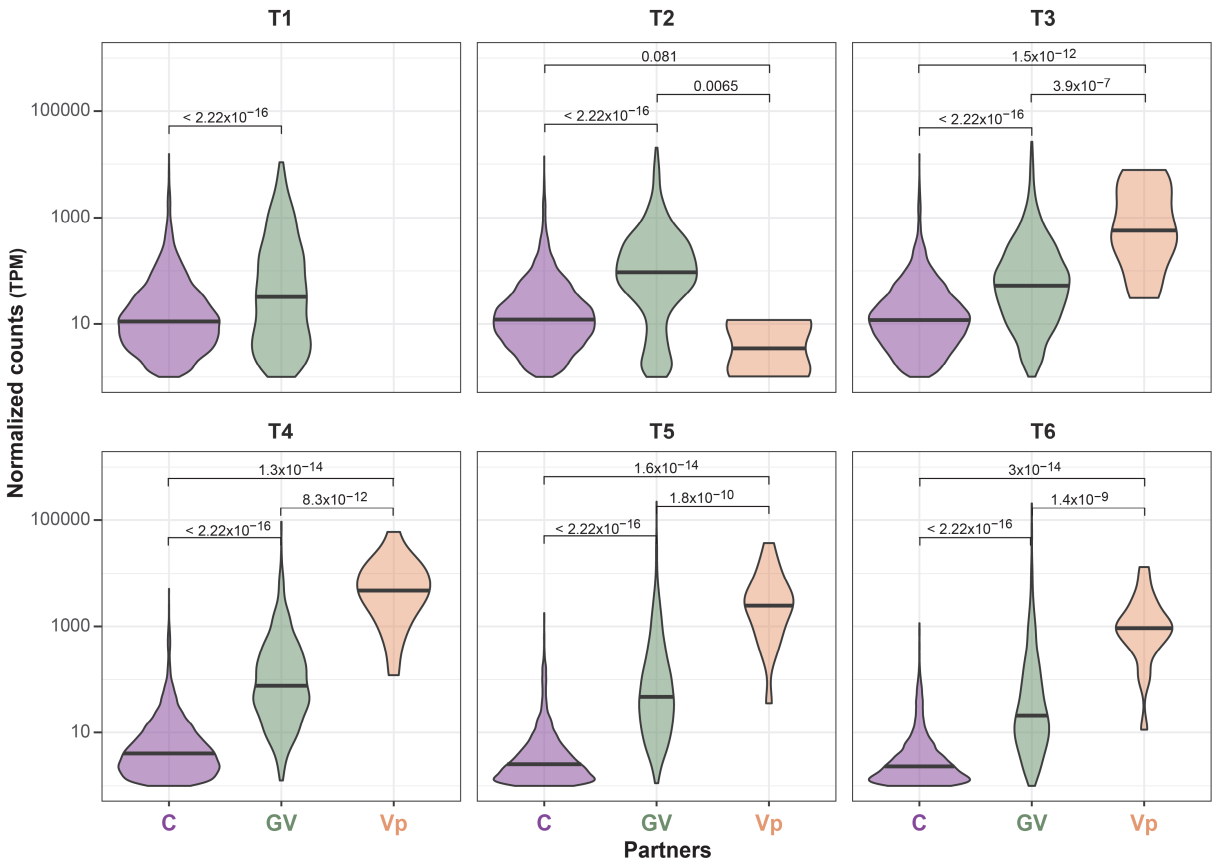

Figure S10. Comparison of zamilon vitis and megavirus chilensis transcriptional activity during infection

Violin plots of normalized counts (TPM) of all genes during giant virus infection in the C+GV+Vp condition. Genes of the host cell (C), giant virus (GV) and virophage (Vp) with expression > 1 TPM were considered. Each panel corresponds to a timepoint (T1 to T6). Black lines show median values. Wilcoxon rank sum test *P*values are noted on top of each comparison. Source data are provided as a Source Data file.

Supplementary Information References

1. Arslan, D., Legendre, M., Seltzer, V., Abergel, C. & Claverie, J.-M. Distant Mimivirus relative with a larger genome highlights the fundamental features of Megaviridae. *Proc. Natl. Acad. Sci. U. S. A.* **108**, 17486–17491 (2011).
2. Krumsiek, J., Arnold, R. & Rattei, T. Gepard: a rapid and sensitive tool for creating dotplots on genome scale. *Bioinforma. Oxf. Engl.* **23**, 1026–1028 (2007).
3. Heinz, S. *et al.* Simple combinations of lineage-determining transcription factors prime cis-regulatory elements required for macrophage and B cell identities. *Mol. Cell* **38**, 576–589 (2010).
4. Emms, D. M. & Kelly, S. OrthoFinder: phylogenetic orthology inference for comparative genomics. *Genome Biol.* **20**, 238 (2019).
