# Supplementary material for: Complex transcriptional regulations of a hyperparasitic quadripartite system in giant viruses infecting protists
Source: Nat Commun. 2024 Oct 9;15:8608. doi: 10.1038/s41467-024-52906-1 (PMC11464507; doi:10.1038/s41467-024-52906-1)
Supplement: Supplementary file 3 — Description of Additional Supplementary Files [file 41467_2024_52906_MOESM3_ESM.pdf]

Description of Additional Supplementary Files

Supplementary Data 1.

Early and late megavirus chilensis giant virus regulatory motifs in promoter regions of megavirus chilensis giant virus, zamilon vitis virophage and megavirus vitis transpoviron

The number of early and/or late giant virus regulatory motifs (see Methods) present in gene promoter regions (100 nt upstream of the start codon) of GV, Vp and Tpv is shown. Percentages indicate the proportion of gene promoters matching a given motif among all genes from a specific cluster.

Supplementary Data 2

A: Number of sequenced read pairs before and after quality control

For each sample, the number of read pairs obtained before and after quality control (QC) is presented. The three different replicates are named R1, R2 and R3. The different conditions and timepoints are defined in Fig. 1A.

B: Number and proportion of read pairs mapped to each partner genome

Number of read pairs mapped to the combined reference genomes using RSEM with bowtie2. Unique and multi mapped filtered read pairs are considered. The different conditions are defined in Fig. 1A. Percentages correspond to the number of mapped read pairs among filtered read pairs. The number and percentage of mapped read pairs is also shown for each individual partner. The three different replicates are named R1, R2 and R3.

Supplementary Data 3

A: Biological processes significantly enriched in A. castellanii genes differentially expressed in T1 compared to Mock

One-sided Fisher Exact test was used to identify enriched biological processes in each group of differentially expressed gene compared to gene universe. Gene universe is defined as all the cellular genes differentially expressed between mock and any timepoint.

B: Biological processes significantly enriched in A. castellanii genes differentially expressed between Mock and any timepoint (T1 to T6)

One-sided Fisher Exact test was used to identify enriched biological processes in each cluster compared to gene universe. Gene universe is defined as all the cellular genes differentially expressed between mock and any timepoint.

C: Functional categories significantly enriched in megavirus chilensis gene expression clusters

One-sided hypergeometric test was used to identify enriched biological processes in each cluster compared to gene universe. Gene universe is defined as all the viral expressed genes during infection.

Supplementary Data 4

A: Biological process functional annotations of A. castellanii genes differentially expressed in T1 compared to Mock

B: Biological process functional annotations of A. castellanii genes differentially expressed between Mock and any timepoint (T1 to T6)

Supplementary Data 5

A: Normalized gene expression of A. castellanii genes in all timepoints and conditions

Gene expression values (TPM) are averaged over the replicates. Conditions are defined in Fig. 1A.

B: Normalized gene expression of megavirus chilensis genes in all timepoints and conditions

Gene expression values (TPM) are averaged over the replicates. Conditions are defined in Fig. 1A.
C: Normalized gene expression of zamilon vitis genes in all timepoints and conditions
Gene expression values (TPM) are averaged over the replicates. Conditions are defined in Fig. 1A.
D: Normalized gene expression of megavirus vitis transpoviron genes in all timepoints and conditions
Gene expression values (TPM) are averaged over the replicates. Conditions are defined in Fig. 1A.

Supplementary Data 6

A: MS-based quantitative proteomic analysis of megavirus chilensis virion without zamilon vitis virophage coinfection
GV virions were purified from cells (single infection). The proteome was analysed by MS-based label-free quantitative proteomics. The results were obtained through the analysis of three biological replicates for each condition. Only proteins identified by MS/MS in two replicates and quantified in the three replicates of one condition were considered. GV proteins are colored in green and ameboa proteins are colorless.
B: MS-based quantitative proteomic analysis of megavirus chilensis virion with zamilon vitis virophage coinfection
GV virions were purified from cells co-infected with Vp. The proteome was analysed by MS-based label-free quantitative proteomics. The results were obtained through the analysis of three biological replicates for each condition. Only proteins identified by MS/MS in two replicates and quantified in the three replicates of one condition were considered. GV proteins are colored in green, Vp proteins in orange, and ameboa proteins are colorless.
C: MS-based quantitative proteomic analysis of megavirus chilensis virions purified from A. castellanii cells co-infected with zamilon vitis virophage or not
Virion proteomes of GV purifed from cells co-infected with Vp or not were analyzed by MS-based label-free quantitative proteomics (three biological replicates per condition). The quantification of proteins (log_2 of filtered, normalized and imputed abundances of the different proteins in the different samples are given in columns K to P) was based on razor and specific peptides (values indicated in column G). Statistical significance was tested using two-sided moderated t-test (limma) for two-by-two sample comparisons ; differentially abundant proteins ("+" in column H) were defined by a log2(fold change) ≥ 1.5 or ≤ -1.5 (column I) and a p-value ≤ 0.01 (column J), allowing to reach a false-discovery rate < 1 % according to the Benjamini-Hochberg estimator.

Supplementary Data 7

Open reading frames identified in terminal inverted repeats of the megavirus vitis transpoviron genome

Supplementary Data 8

A: Differentially expressed A. castellanii genes during co-infections vs single infection
The table shows the basemean, logFC and ajusted FDR Pvalue (two-sided Wald test) calculated by Deseq2. Differential expression analyses comparing two conditions at a single time point were conducted using both DESeq2 and edgeR. Genes identified by the both methods are considered differentially expressed, with their values highlighted in red (overexpression) or blue (underexpression).
B: Differentially expressed megavirus chilensis genes during co-infections vs single infections
The table shows the basemean, logFC and ajusted FDR Pvalue (two-sided Wald test) calculated by Deseq2. Differential expression analyses comparing two conditions at a single time point were conducted using both DESeq2 and edgeR. Genes identified by the both methods are considered differentially expressed, with their values highlighted in red (overexpression) or blue (underexpression).
C: Differentially expressed zamilon vitis genes during co-infections with megavirus vitis transpoviron or not
The table shows the basemean, logFC and ajusted FDR Pvalue (two-sided Wald test) calculated by Deseq2.

D: Differentially expressed megavirus vitis transpoviron genes during co-infections with zamilon vitis virophage or not

The table shows the basemean, logFC and ajusted FDR Pvalue (two-sided Wald test) calculated by Deseq2. Differential expression analyses comparing two conditions at a single time point were conducted using both DESeq2 and edgeR. Genes identified by the both methods are considered differentially expressed, with their values highlighted in red (overexpression) or blue (underexpression).

Supplementary Data 9

MS-based quantitative proteomic analysis of zamilon vitis virion

Vp virions were purified from cells co-infected with GV and Tpv. Their proteomes were analysed by MS-based label-free quantitative proteomics. The results were obtained through the analysis of two biological replicates. Only proteins detected in the two replicates were considered. Vp proteins are colored in orange, GV proteins in green, Tpv proteins in blue, and ameboa proteins are colorless. For each protein, the copy number was estimated by a rule of three using the normalised iBAQ values and the copy number of the Vp major capsid protein (za3_12); only proteins with an estimated copy number above 1 are presented.

Supplementary Data 10

Accession numbers of samples

Supplementary Data 11

Genomic coordinates and annotations of *Acanthamoeba castellanii* genes
